# Thymol Chemotype *Thymus vulgaris* L. Hydrolate: Volatile Chemical Fraction Composition and Antimicrobial Activity Against Bacterial and Fungal Pathogens

**DOI:** 10.1002/cbdv.71425

**Published:** 2026-06-15

**Authors:** Stefania Garzoli, Joel Horacio Elizondo‐Luevano, Anis Ben Hsouna, Rania Ben Saad, Miroslava Kačániová

**Affiliations:** ^1^ Department of Chemistry and Technologies of Drug Sapienza University Rome Italy; ^2^ Laboratory of Natural Sciences Biomolecular Innovation Group Faculty of Agronomy Universidad Autónoma de Nuevo León Cd. Gral. Escobedo Nuevo León Mexico; ^3^ Laboratory of Biotechnology and Plant Improvement Centre of Biotechnology of Sfax Sfax Tunisia; ^4^ Department of Environmental Sciences and Nutrition Higher Institute of Applied Sciences and Technology of Mahdia University of Monastir Monastir Tunisia; ^5^ Institute of Horticulture Faculty of Horticulture and Landscape Engineering Slovak University of Agriculture Nitra Slovakia; ^6^ School of Medical & Health Sciences VIZJA University Warszawa Poland

**Keywords:** *Thymus vulgaris* hydrolate, HS‐GC‐MS, antimicrobial activity, natural preservative, bacteria, filamentous fungi, yeasts

## Abstract

The increasing prevalence of antimicrobial resistance necessitates the development of alternative antimicrobial agents derived from natural sources. This study evaluated the volatile chemical profile and antimicrobial activity of a thymol‐containing hydrolate obtained from *Thymus vulgaris* L. against a panel of 14 clinically and economically relevant microorganisms. Headspace‐gas chromatography–mass spectrometry analysis revealed thymol (79.9%) and linalool (17.4%) as the predominant constituents of the hydrolate. Antimicrobial activity was assessed using disc diffusion and broth microdilution assays to determine inhibition zones and minimum inhibitory concentrations. The hydrolate exhibited pronounced activity against Gram‐positive bacteria, with the largest inhibition zones observed for *Staphylococcus aureus* and the lowest MIC values for the same strain (MIC_50_ = 5.765 mg/mL; MIC_90_ = 6.341 mg/mL). Gram‐negative bacteria displayed moderate susceptibility, with MIC_50_ values ranging from 13.605 to 15.946 mg/mL. Yeasts showed lower susceptibility (*Candida albicans* MIC_50_ = 25.450 mg/mL), while filamentous fungi exhibited the highest resistance (*Botrytis cinerea* MIC_50_ = 46.013 mg/mL). Overall antimicrobial efficacy followed the pattern: Gram‐positive bacteria > Gram‐negative bacteria > yeasts > filamentous fungi. These findings demonstrate that thymol hydrolate possesses substantial antimicrobial potential, particularly against Gram‐positive pathogens, and may represent a promising natural alternative to synthetic antimicrobial agents for applications in food preservation and cosmetic formulations.

## Graphical abstract

Phytochemistry and bioactivity of thymol chemotype *Thymus vulgaris* L. hydrolate.

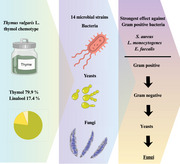

## 1 Introduction

Antimicrobial resistance is recognized as one of the most serious challenges in modern medicine and food safety. The excessive and often inappropriate use of synthetic antimicrobial agents has accelerated the emergence of multidrug‐resistant pathogens, thereby intensifying the need for alternative antimicrobial strategies derived from natural sources. In this context, essential oils and their aqueous by‐products, known as hydrolates or hydrosols, have attracted increasing scientific interest due to their broad‐spectrum antimicrobial activity, biodegradability, and comparatively lower toxicity [1, 2].

Hydrolates are produced as secondary products during steam distillation or hydrodistillation of aromatic plants for essential oil extraction. Unlike essential oils, which are composed of highly concentrated volatile compounds, hydrolates consist predominantly of water‐soluble aromatic constituents and small quantities of dissolved essential oil components, typically ranging from 0.02% to 0.2% [3]. Although hydrolates contain lower concentrations of bioactive compounds, numerous studies have demonstrated their notable antimicrobial activity [3, 4]. Moreover, hydrolates are often considered to have a favorable safety profile, although their regulatory status may vary depending on application and region, making them suitable for applications in the food, pharmaceutical, and cosmetic industries [5].

*Thymus vulgaris* L. (common thyme) is an aromatic plant belonging to the Lamiaceae family and is widely used in Mediterranean cuisine as well as in traditional medicine. Thyme essential oil is characterized by a high content of phenolic monoterpenes, predominantly thymol (30%–70%) and carvacrol (3%–15%), which are largely responsible for its strong antimicrobial properties [6]. Thymol exerts antimicrobial effects through multiple mechanisms, including disruption of microbial cell membranes, increased membrane permeability, and interference with cellular energy metabolism [5, 7].

Despite extensive research on thyme essential oil, hydrolates derived from *Thymus vulgaris* L. have received comparatively less attention in the scientific literature [4]. Given their milder sensory profile, lower toxicity, and ease of application, a systematic evaluation of the antimicrobial efficacy of thyme hydrolates is essential for their potential incorporation into modern food preservation systems [3].

Foodborne and spoilage microorganisms represent major threats to public health, food quality, and food security. Gram‐positive bacteria such as *Staphylococcus aureus*, *Listeria monocytogenes*, and *Enterococcus faecalis* are frequently associated with foodborne diseases and contamination [8]. Similarly, Gram‐negative pathogens, including *Escherichia coli*, *Salmonella enterica*, and *Yersinia enterocolitica* are among the most common causative agents of food contamination and outbreaks [9]. In addition, fungal pathogens, particularly yeasts of the genus *Candida* and filamentous fungi such as *Botrytis cinerea* and *Fusarium solani* cause significant economic losses in agriculture and postharvest storage.

The aim of the present study was to evaluate the antimicrobial activity of a thymol‐containing hydrolate against a panel of clinically and economically relevant microorganisms, including Gram‐positive and Gram‐negative bacteria, yeasts, and filamentous fungi. Minimum inhibitory concentrations (MIC_50_ and MIC_90_) were determined using the broth microdilution method. Furthermore, the correlation between disc diffusion assay results and MIC values was assessed to evaluate the suitability of disc diffusion as a rapid screening method. Finally, the antimicrobial efficacy of thymol hydrolate was compared with previously published data on other plant‐derived hydrolates to establish its relative potency. This study contributes to the growing body of evidence supporting plant hydrolates as sustainable and effective alternatives to conventional antimicrobial agents for applications in food preservation and cosmetic formulations.

## 2 Material and Methods

### 2.1 Hydrolate Sample

A thymol‐containing hydrolate (thyme hydrosol) was obtained from HANUS s.r.o. (Nitra, Slovakia; EAN: 8588008728161). According to the manufacturer's specifications, the product was produced by steam distillation of flowering aerial parts of *Thymus vulgaris* L. (Lamiaceae) of the thymol chemotype. The hydrolate consisted of a 100% aqueous distillate without the addition of preservatives or synthetic additives. The product was stored at 4°C in its original dark glass bottle until further use.

### 2.2 Volatile Chemical Composition of *Thymus vulgaris* L. Hydrolate

The chemical characterization of the volatile fraction of hydrolate was performed by the use of a Perkin–Elmer Headspace Turbo‐matrix 40 autosampler (Waltham, MA, USA) connected directly to the GC–MS apparatus. About 2 mL of *Thymus vulgaris* L. were placed in 20 mL vial sealed with headspace PTFE‐coated silicone rubber septa and cap. The operative parameters were optimized and following Garzoli et al. [10] with some minor modifications. The GC capillary column housed in the oven was a Varian Factor Four VF‐5 (60 m × 0.32 mm ID, DF = 1.0 µm) and helium was the carrier gas at a flow rate of 1 mL/min. The temperature program was set as follows: 60°C up to 220°C for 20 min at a rate of 6°C min^−1^. For the identification of components, a match between the mass spectra and those found in the databases of the NIST and Wiley libraries was performed. Furthermore, the linear retention indices (LRIs) were calculated for each component and compared with those obtained by use of a mixture of *n*‐alkanes (C_8_–C_30_) injected under the same conditions. The relative amounts expressed as average percentages were calculated by peak area normalization from GC‐FID chromatograms. All analyses were conducted in triplicate.

### 2.3 Microorganisms and Cultivation Conditions

The antimicrobial activity of thymol hydrolate was evaluated against a panel of selected bacterial and fungal strains obtained from the Czech Collection of Microorganisms (CCM). The bacterial panel included Gram‐positive species *E. faecalis* (CCM 4224), *L. monocytogenes* (CCM 4699), and *Staphylococcus aureus* subsp. *aureus* (CCM 4423), as well as Gram‐negative species *E. coli* (CCM 3953), *S. enterica* subsp. *enterica* (CCM 3807), and *Y. enterocolitica* (CCM 7204ᵀ). The fungal panel consisted of opportunistic yeasts *Candida albicans* (CCM 8136), *C. glabrata* (CCM 8270), *C. krusei* (CCM 8271), *C. parapsilosis* (CCM 8260), and *C. tropicalis* (CCM 8264), as well as filamentous fungi *B. cinerea* (CCM F‐314), *F. solani* (CCM 8014), and *Trichoderma harzianum* (CCM F‐470).

Bacterial strains were cultivated in Mueller–Hinton Broth (MHB; Oxoid, Basingstoke, UK), yeasts in Sabouraud dextrose broth (SDB; Oxoid), and filamentous fungi in potato dextrose broth (PDB; Oxoid). Incubation was performed at 37°C for bacteria and at 25°C for yeasts and filamentous fungi for 24 h (bacteria and yeasts) or 5–7 days (filamentous fungi). Prior to antimicrobial testing, microbial suspensions were adjusted to the 0.5 McFarland turbidity standard, corresponding to approximately 1.5 × 10^8^ CFU/mL, to ensure uniform inoculum density across all experiments.

### 2.4 Agar Disc Diffusion Assay

Preliminary screening of the antimicrobial activity of thymol hydrolate was conducted using the agar disc diffusion method. Sterile paper discs (6 mm diameter) were impregnated with 10 µL of hydrolate and placed onto Mueller–Hinton Agar (MHA) plates inoculated with bacterial suspensions, Sabouraud Dextrose Agar (SDA) plates inoculated with yeasts, or potato dextrose agar (PDA) plates inoculated with filamentous fungi. Plates were incubated at 37°C for bacterial strains and at 25°C for yeasts and filamentous fungi for 24 h (bacteria and yeasts) or 5–7 days (filamentous fungi). Following incubation, the diameters of inhibition zones surrounding each disc, including the 6 mm disc diameter, were measured and recorded. Sterile distilled water served as the negative control. Positive controls included cefoxitin for Gram‐positive bacteria, gentamicin for Gram‐negative bacteria, and amphotericin B (10 µg) for yeasts and filamentous fungi (Oxoid, Basingstoke, UK). All assays were performed in triplicate.

### 2.5 Determination of Minimum Inhibitory Concentrations (MIC_50_ and MIC_90_)

MIC_50_ and MIC_90_ of thymol hydrolate were determined using the broth microdilution method in sterile 96‐well microtiter plates. Each well contained 100 µL of microbial suspension adjusted to the 0.5 McFarland standard and 100 µL of hydrolate serially diluted two‐fold in Mueller–Hinton Broth (MHB) for bacteria or Sabouraud Dextrose Broth (SDB) for yeasts and filamentous fungi. Hydrolate concentrations ranged from 100 to 0.049 mg/mL across 12 dilution steps.

Negative controls consisted of broth containing hydrolate without microbial inoculum, while positive controls consisted of inoculated broth without hydrolate. Plates were incubated for 24 h at 37°C for bacteria and at 25°C for yeasts, or for 5–7 days at 25°C for filamentous fungi. Microbial growth was quantified by measuring absorbance at 570 nm using a microplate reader. MIC_50_ and MIC_90_ values were defined as the lowest hydrolate concentrations resulting in 50% and 90% inhibition of microbial growth, respectively, compared to untreated controls. MIC_50_ and MIC_90_ values were calculated using probit analysis based on the relationship between the logarithm of hydrolate concentration and the percentage of microbial growth inhibition. All measurements were performed in triplicate.

## 3 Results

### 3.1 Chemical Composition of *Thymus vulgaris* L. Hydrolate

**FIGURE 1:**
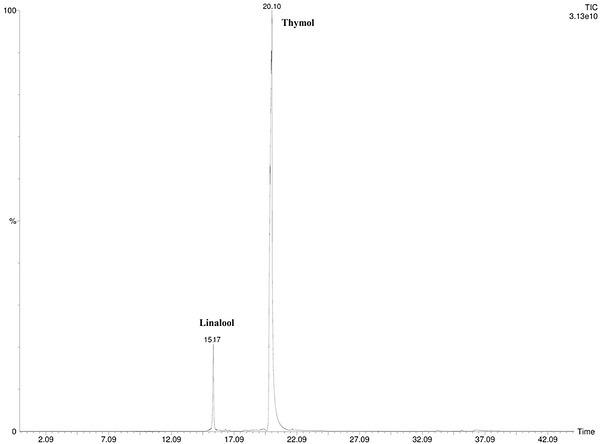
GC–MS chromatogram of *Thymus vulgaris* L. hydrolate.

The chemical composition of the thymol‐containing hydrolate was determined by headspace‐gas chromatography–mass spectrometry (HS‐GC–MS) analysis. The results are presented in Table 1. The hydrolate was characterized by a high thymol content (79.7%), followed by linalool (17.4%) as the second major component. Minor constituents included *α*‐terpineol (1.0%), carvacrol (0.7%), terpinen‐4‐ol (0.5%), 1,8‐cineole (0.3%), and camphor (0.2%). Trace amounts of β‐myrcene, limonene, and *α*‐ocimene were also detected. The GC–MS chromatogram has reported in Figure 1.

**TABLE 1:** Chemical composition of *Thymus vulgaris* L. hydrolate determined by HS‐GC–MS analysis.

Nos.	Compounds	LRI ^calc^	LRI ^lit^	%^a^
1	*β*‐Myrcene	979	982	tr
2	Limonene	1028	1031	tr
3	1,8‐Cineole	1035	1033	0.3 ± 0.02
4	*α*‐Ocimene	1038	1042	tr
5	Linalool	1079	1082	17.4 ± 0.15
6	Camphor	1141	1139	0.2 ± 0.02
7	Terpinen‐4‐ol	1185	1182	0.5 ± 0.03
8	*α*‐Terpineol	1192	1198	1.0 ± 0.05
9	Thymol	1263	1267	79.7 ± 6.20
10	Carvacrol	1295	1297	0.7 ± 0.03
	**SUM** ^b^			99.8

The components are reported according to their elution order on a polar column; LRI ^calc^: LRI measured on a polar column; LRI ^lit^: LRI from literature; tr: percentage mean values ≤ 0.1%.

^a^ Percentage mean values ± SD.

^b^ Sum of the mean percentage values of all detected compounds.

### 3.2 Antimicrobial Activity by Disc Diffusion Method

The antimicrobial activity of thymol hydrolate was evaluated using the disc diffusion method against 14 microbial strains, including Gram‐positive bacteria, Gram‐negative bacteria, yeasts, and filamentous fungi. The results, expressed as inhibition zone diameters (including the 6 mm disc), are presented in Table 2.

**TABLE 2:** Antimicrobial activity assessed by the disc diffusion method.

Microorganisms	Inhibition zone of thymol hydrolate (mm)	Inhibition zone of ATB (mm)
Gram‐negative bacteria		
*Escherichia coli* CCM 3953	10.67 ± 0.58	29.67 ± 0.58
*Salmonella enterica* CCM 3807	9.67 ± 0.58	32.33 ± 0.58
*Yersinia enterocolitica* CCM 7204T	11.67 ± 0.58	30.33 ± 0.58
Gram‐positive bacteria		
*Enterococcus faecalis* CCM 4224	15.33 ± 0.58	31.33 ± 0.58
*Listeria monocytogenes* CCM 4699	14.33 ± 0.58	31.67 ± 0.58
*Staphylococcus aureus* CCM 4423	15.67 ± 0.58	31.67^j^ ± 0.58
Yeast		
*Candida albicans* CCM 8136	7.33 ± 0.58	30.33 ± 0.58
*Candida glabrata* CCM 8270	7.33 ± 0.58	30.67 ± 0.58
*Candida krusei* CCM 8271	6.67 ± 0.58	29.33 ± 0.58
*Candida parapsilosis* CCM 8260	8.33 ± 0.58	26.33 ± 0.58
*Candida tropicalis* CCM 8264	7.33 ± 0.58	26.33 ± 0.58
Fungi		
*Botrytis cinerea* F‐314	5.67 ± 0.58	23.33 ± 0.58
*Fusarium solani* CCM 8014	4.67 ± 0.58	24.33 ± 0.58
*Trichoderma harzianum* CCM F‐470	6.67 ± 0.58	25.33 ± 0.58

*Note*: Data represents the mean (± standard deviation) of 3 samples.

Thymol hydrolate exhibited the strongest activity against Gram‐positive bacteria. The largest inhibition zones were observed for *S. aureus* (16.33 mm), followed by *E. faecalis* (15.33 mm) and *L. monocytogenes* (14.33 mm). Among Gram‐negative bacteria, moderate inhibition zones were recorded, with *Y. enterocolitica* showing the highest susceptibility (9.67 mm), followed by *S. enterica* (8.67 mm) and *E. coli* (8.33 mm).

Yeasts demonstrated relatively low susceptibility to thymol hydrolate. Inhibition zone diameters ranged from 5.33 mm (*C. krusei* and *C. tropicalis*) to 6.33 mm (*C. parapsilosis*). *C. albicans* and *C. glabrata* exhibited identical inhibition zones (5.67 mm). Filamentous fungi showed the weakest response to thymol hydrolate, with inhibition zones ranging from 4.67 mm (*T. harzianum*) to 5.67 mm (*B. cinerea*). *F. solani* exhibited an intermediate inhibition zone of 5.33 mm.

Positive controls amphotericin B for yeasts and filamentous fungi, gentamicin for Gram‐negative bacteria, and cefoxitin for Gram‐positive bacteria produced substantially larger inhibition zones (23.33–32.33 mm) than thymol hydrolate, confirming the expected antimicrobial activity of the reference agents.

### 3.3 Minimum Inhibitory Concentrations (MIC_50_ and MIC_90_)

The minimum inhibitory concentrations (MIC_50_ and MIC_90_) of thymol hydrolate were determined using the broth microdilution method. MIC_50_ values ranged from 5.765 mg/mL (*S. aureus*) to 46.013 mg/mL (*B. cinerea*), while MIC_90_ values ranged from 6.341 mg/mL (*S. aureus*) to 50.614 mg/mL (*B. cinerea*). The results are presented in Table 3.

**TABLE 3:** Minimal inhibitory concentration in mg/mL.

Microorganisms	MIC_50_	MIC_90_
Gram‐negative bacteria		
*Escherichia coli* CCM 3953	7.386 ± 0.612	8.347 ± 0.692
*Salmonella enterica* CCM 3807	6.714 ± 0.558	7.587 ± 0.631
*Yersinia enterocolitica* CCM 7204T	12.662 ± 0.166	14.308 ± 0.187
Gram‐positive bacteria		
*Enterococcus faecalis* CCM 4224	6.280 ± 0.189	7.097 ± 0.213
*Listeria monocytogenes* CCM 4699	10.133 ± 0.195	11.451 ± 0.221
*Staphylococcus aureus* CCM 4423	5.442 ± 0.185	6.149 ± 0.209
Yeast		
*Candida albicans* CCM 8136	20.099 ± 0.028	22.713 ± 0.032
*Candida glabrata* CCM 8270	22.228 ± 0.651	25.118 ± 0.736
*Candida krusei* CCM 8271	18.459 ± 0.040	20.859 ± 0.045
*Candida parapsilosis* CCM 8260	19.017 ± 0.819	21.489 ± 0.926
*Candida tropicalis* CCM 8264	31.191 ± 0.114	35.246 ± 0.129
Fungi		
*Botrytis cinerea* F‐314	37.537 ± 0.494	42.416 ± 0.559
*Fusarium solani* CCM 8014	34.744 ± 1.117	39.261 ± 1.262
*Trichoderma harzianum* CCM F‐470	27.521 ± 0.578	31.099 ± 0.653

*Note*: Data represents the mean (± standard deviation) of 3 samples.

Among Gram‐positive bacteria, thymol hydrolate exhibited the highest activity against *S. aureus* (MIC_50_ = 5.765 mg/mL; MIC_90_ = 6.341 mg/mL), followed by *L. monocytogenes* (MIC_50_ = 6.714 mg/mL; MIC_90_ = 7.386 mg/mL) and *E. faecalis* (MIC_50_ = 7.638 mg/mL; MIC_90_ = 8.402 mg/mL).

Gram‐negative bacteria showed higher MIC values than Gram‐positive strains. The lowest MIC values among Gram‐negative bacteria were observed for *Y. enterocolitica* (MIC_50_ = 13.605 mg/mL; MIC_90_ = 14.966 mg/mL), followed by *S. enterica* (MIC_50_ = 13.727 mg/mL; MIC_90_ = 15.100 mg/mL). *E. coli* exhibited the highest MIC values among the tested bacterial strains (MIC_50_ = 15.946 mg/mL; MIC_90_ = 17.540 mg/mL).

Yeasts demonstrated substantially higher MIC values than bacteria, indicating lower susceptibility to thymol hydrolate. Among *Candida* species, *C. albicans* exhibited the lowest MIC values (MIC_50_ = 25.450 mg/mL; MIC_90_ = 27.996 mg/mL), whereas *C. tropicalis* showed the highest resistance (MIC_50_ = 41.422 mg/mL; MIC_90_ = 45.564 mg/mL). Intermediate MIC_50_ values were observed for *C. glabrata* (27.591 mg/mL), *C. krusei* (31.776 mg/mL), and *C. parapsilosis* (37.710 mg/mL).

Filamentous fungi exhibited the highest MIC values among all tested microorganisms. *T. harzianum* showed the lowest MIC values within this group (MIC_50_ = 35.477 mg/mL; MIC_90_ = 39.025 mg/mL), followed by *F. solani* (MIC_50_ = 43.106 mg/mL; MIC_90_ = 47.417 mg/mL). *B. cinerea* demonstrated the highest resistance to thymol hydrolate (MIC_50_ = 46.013 mg/mL; MIC_90_ = 50.614 mg/mL).

Overall, the antimicrobial efficacy of thymol hydrolate followed the pattern: Gram‐positive bacteria > Gram‐negative bacteria > yeasts > filamentous fungi.

## 4 Discussion

### 4.1 Chemical Composition and Chemotype Confirmation

GC–MS analysis of the thymol‐containing hydrolate revealed thymol (79.9%) as the predominant component, confirming that the *Thymus vulgaris* L. material used in this study belonged to the thymol chemotype. This observation is consistent with previous reports on thyme essential oils, in which thymol concentrations typically range from 30% to 70% [1, 6]. The notably high thymol content (79.9%) detected in the present hydrolate indicates efficient transfer of phenolic monoterpenes into the aqueous phase during steam distillation. Linalool (17.4%) was identified as the second major component, a compositional feature characteristic of thymol chemotype thyme. The co‐occurrence of thymol and linalool has been widely reported in *Thymus vulgaris* essential oils and may enhance overall antimicrobial efficacy through additive or synergistic interactions [7]. Minor constituents such as *α*‐terpineol (1.0%), terpinen‐4‐ol (0.5%), and 1,8‐cineole (0.3%) are commonly detected in thyme‐derived products and are known to exhibit antimicrobial activity, although generally at lower potency than thymol [5].

The very low carvacrol content (0.7%) detected in the analyzed hydrolate further supports the classification of the source plant material as a thymol chemotype and clearly differentiates it from carvacrol‐dominant thyme products. This chemotype specificity is particularly important for standardization, reproducibility, and quality control in commercial applications, as different chemotypes are associated with distinct biological activities and safety profiles [11, 12].

### 4.2 Antimicrobial Activity Against Gram‐Positive Bacteria

Thymol hydrolate exhibited pronounced antimicrobial activity against Gram‐positive bacteria, with *S. aureus* showing the highest susceptibility (MIC_50_ = 5.765 mg/mL; inhibition zone 16.33 mm). This finding is particularly relevant given that *S. aureus* is a major cause of foodborne illnesses and nosocomial infections, and that the increasing prevalence of methicillin‐resistant *S. aureus* (MRSA) strains poses significant challenges to conventional antimicrobial therapies [13, 14]. Recent systematic reviews have confirmed MRSA contamination in retail meat and food products globally, with foodborne MRSA strains often harboring multiple virulence factors that facilitate horizontal dissemination of antimicrobial resistance genes [15].

The antimicrobial activity observed against *S. aureus* is comparable to that reported for previously studied plant‐derived hydrolates, such as concentrated clove hydrolate [16]. For example, concentrated clove hydrolate obtained by 50‐fold solid‐phase extraction has been reported to exhibit MIC values of 4–8 mg/mL against *S. aureus*, with inhibition zones ranging from 15.5 to 15.8 mm [16]. In contrast, the non‐concentrated thymol hydrolate evaluated in the present study achieved similar inhibitory effects (inhibition zone 16.33 mm; MIC_50_ = 5.765 mg/mL), indicating a relatively high antimicrobial potency despite the absence of any concentration step.

*L. monocytogenes* (MIC_50_ = 6.714 mg/mL) and *E. faecalis* (MIC_50_ = 7.638 mg/mL) also exhibited high susceptibility to thymol hydrolate. *L. monocytogenes* is of particular concern in ready‐to‐eat foods due to its psychrotrophic growth ability and high mortality rates among vulnerable populations [17, 18]. The strong inhibitory effect observed against this pathogen highlights the potential applicability of thymol hydrolate in food preservation systems, especially for refrigerated products where conventional thermal treatments are unsuitable.

The antimicrobial mechanism of thymol against Gram‐positive bacteria involves multiple cellular targets. Due to its lipophilic nature, thymol can integrate into bacterial cell membranes, leading to disruption of membrane integrity, dissipation of the proton motive force (including membrane potential hyperpolarization and pH gradient collapse), and depletion of intracellular ATP reserves through blockade of ATP synthesis pathways [19, 20]. In addition, thymol has been reported to inhibit essential bacterial enzymes and interfere with protein synthesis, contributing to its broad‐spectrum bactericidal activity [21].

The presence of linalool (17.5%) may further contribute to the antimicrobial efficacy of the hydrolate. Linalool possesses intrinsic antimicrobial activity through mechanisms including membrane depolarization, increased membrane permeability, disruption of ATP synthesis, and interference with respiratory metabolism [22, 23]. Linalool may enhance thymol action by increasing bacterial membrane permeability, thereby facilitating thymol penetration into bacterial cells and exhibiting synergistic or additive antimicrobial effects [24]. Such multicomponent interactions are characteristic of plant‐derived antimicrobials and may offer advantages over single‐compound synthetic agents, including a reduced likelihood of resistance development.

### 4.3 Antimicrobial Activity Against Gram‐Negative Bacteria

Gram‐negative bacteria exhibited moderate susceptibility to thymol hydrolate, with MIC_50_ values approximately two‐ to three‐fold higher than those observed for Gram‐positive bacteria. Among the tested Gram‐negative strains, *Y. enterocolitica* showed the lowest MIC_50_ value (13.605 mg/mL), followed by *S. enterica* (13.727 mg/mL) and *E. coli* (15.946 mg/mL).

The reduced susceptibility of Gram‐negative bacteria compared to Gram‐positive strains is consistent with well‐established structural differences in bacterial cell envelopes. Gram‐negative bacteria possess an outer membrane rich in lipopolysaccharides (LPS), which functions as an effective permeability barrier limiting the penetration of hydrophobic antimicrobial compounds such as thymol [25, 26]. The LPS molecules are located exclusively in the outer leaflet of the asymmetric outer membrane, creating a highly ordered structure that significantly reduces permeability to hydrophobic antibiotics and other lipophilic agents [27]. This additional barrier must be overcome before thymol can reach the cytoplasmic membrane and exert its antimicrobial effect.

Despite this intrinsic resistance mechanism, the MIC values obtained in the present study (13.6–15.9 mg/mL) remain within a potentially applicable range for food‐related applications. Previous studies on thyme essential oil have reported substantially lower MIC values (0.10–0.45 mg/mL) against Gram‐negative bacteria, including *Salmonella* spp. and *E. coli* [28, 29], indicating approximately 30‐ to 150‐fold greater potency compared to thymol hydrolate. The substantial difference in antimicrobial activity between essential oil and hydrolate is expected, given the markedly lower concentration of volatile antimicrobial compounds in hydrolates (typically 0.02%–0.2%) compared to essential oils.

The observed activity against *S. enterica* is of particular relevance for food safety, as this pathogen is among the most frequent causes of foodborne gastroenteritis worldwide, with an estimated 93.8 million cases annually and 155,000 deaths globally [30, 31]. Recent surveillance data indicate that *Salmonella* accounted for 19.6% of all foodborne outbreaks in the European Union in 2023, representing the largest proportion among bacterial foodborne pathogens [32]. These results suggest that thymol hydrolate could be considered for use in washing solutions for fresh produce or as a supplementary natural sanitizer in food processing environments to reduce *Salmonella* contamination.

Notably, disc diffusion assays revealed only minor differences in inhibition zone diameters among Gram‐negative bacteria (8.33–9.67 mm), whereas MIC determination demonstrated greater variability in susceptibility (13.6–15.9 mg/mL). This discrepancy underscores the importance of quantitative methods such as MIC determination in addition to qualitative disc diffusion screening, as the diffusion behavior of hydrolate constituents in agar media may not accurately reflect their true antimicrobial potency.

### 4.4 Antifungal Activity Against Yeasts

Yeast species exhibited markedly lower susceptibility to thymol hydrolate compared to bacteria, with MIC_50_ values ranging from 25.450 mg/mL (*C. albicans*) to 41.422 mg/mL (C. tropicalis). This reduced susceptibility is consistent with fundamental structural differences between prokaryotic bacteria and eukaryotic fungi. Fungal cells possess a rigid cell wall composed primarily of chitin and β‐glucans [33], as well as ergosterol‐containing plasma membranes [34], which collectively confer increased resistance to antimicrobial agents relative to bacterial cells [35].

Among the tested *Candida* species, *C. albicans* exhibited the lowest MIC_50_ value (25.450 mg/mL). This observation is of particular relevance given that *C. albicans* is the most common opportunistic fungal pathogen in humans, responsible for infections ranging from superficial mucosal candidiasis to invasive systemic disease in immunocompromised individuals [36, 37]. The moderate antifungal activity observed suggests that thymol hydrolate may be suitable for use in topical formulations or as a natural preservative in cosmetic products to limit fungal contamination.

The susceptibility pattern observed among *Candida* species (*C. albicans* < *C. glabrata* < *C. krusei* < *C. parapsilosis* < *C. tropicalis*) indicates species‐specific differences in intrinsic resistance mechanisms. In particular, *C. tropicalis*, which exhibited the highest MIC_50_ value (41.422 mg/mL), is known for its strong biofilm‐forming capacity and production of multiple virulence factors [38], which may contribute to its reduced susceptibility to antimicrobial agents.

The antifungal activity of thymol is primarily attributed to its interaction with ergosterol, the major sterol component of fungal cell membranes, which disrupts membrane integrity, increases permeability, and results in leakage of intracellular components [34, 39]. Thymol has also been reported to interfere with fungal cell wall synthesis and inhibit germ tube formation, an important virulence factor in *Candida* species [40].

MIC determination provides a more reliable quantitative measure of antifungal activity than disc diffusion assays, as differences in diffusion behavior of hydrolate constituents in agar media can significantly affect inhibition zone measurements.

### 4.5 Antifungal Activity Against Filamentous Fungi

Filamentous fungi exhibited the lowest susceptibility to thymol hydrolate among all tested microorganisms, with MIC_50_ values ranging from 35.477 mg/mL (*T. harzianum*) to 46.013 mg/mL (*B. cinerea*). This reduced susceptibility can be attributed to the complex multicellular organization of filamentous fungi, their thick cell walls composed of multiple polysaccharide layers [41], and their capacity to produce extracellular enzymes and secondary metabolites that may reduce the effectiveness of antimicrobial compounds [42].

*B. cinerea*, which exhibited the highest MIC_50_ value (46.013 mg/mL), is an economically important phytopathogen responsible for gray mold disease in a wide range of crops, including grapes, strawberries, and tomatoes [43]. The rapid development of resistance to synthetic fungicides by this pathogen has stimulated interest in natural antifungal alternatives [44]. Although the MIC values obtained in the present study are relatively high, published data indicate that thyme essential oils typically exhibit MIC values in the range of 0.085–0.3 mg/mL against *B. cinerea* [45], demonstrating substantially higher antifungal potency. This difference is expected, given the markedly lower concentration of bioactive volatile compounds in hydrolates compared to essential oils [46].

*F. solani* (MIC_50_ = 43.106 mg/mL) is another agriculturally relevant pathogen causing root rot and wilt diseases in numerous crops, as well as opportunistic infections in immunocompromised individuals [47]. Previous studies have reported MIC values of 0.085‐0.3 mg/mL for thyme, oregano, and marjoram essential oils against *Fusarium* species [45], further emphasizing the greater antifungal activity of essential oils relative to hydrolates.

In contrast, *T. harzianum* (MIC_50_ = 35.477 mg/mL), which is widely used as a biological control agent against plant pathogens [48], showed comparatively lower susceptibility. This observation suggests that, if thymol hydrolate were to be applied in agricultural environments, selective application strategies would be required to avoid potential interference with beneficial fungal species.

Disc diffusion assays revealed very small inhibition zones for filamentous fungi (4.67–5.67 mm), only marginally exceeding the disc diameter (6 mm). This limited inhibition may be attributed to several factors, including the restricted diffusion of hydrophobic thymol through aqueous agar media, rapid hyphal growth that may outpace antimicrobial diffusion [49], and possible interactions between thymol and agar components. These findings further support the use of MIC determination as a more reliable quantitative approach for assessing antifungal activity than disc diffusion assays [50].

Recent work by Menicucci et al. [4] demonstrated that thyme essential oil and hydrolate inhibited fungal growth on paper substrates, suggesting potential applications in the preservation of cellulose‐based materials. Although the present study focused on food‐related and clinically relevant fungi, these observations indicate that thymol hydrolate may also have broader applicability in material preservation and cultural heritage conservation.

### 4.6 Correlation Between Disc Diffusion and MIC Methods

An important observation of this study is the variable relationship between inhibition zone diameters obtained by the disc diffusion method and MIC values determined by broth microdilution across different microbial groups. For Gram‐positive bacteria, an inverse relationship was observed, whereby larger inhibition zones were generally associated with lower MIC values (e.g., *S. aureus*: 16.33 mm, MIC_50_ = 5.765 mg/mL; *L. monocytogenes*: 14.33 mm, MIC_50_ = 6.714 mg/mL). This pattern indicates a relatively good level of agreement between the two methods for Gram‐positive bacteria, consistent with established guidelines for conventional antimicrobial susceptibility testing [51].

In contrast, for Gram‐negative bacteria and fungi, the relationship between inhibition zone diameters and MIC values was weak or inconsistent. For example, *E. coli*, *S. enterica*, and *Y. enterocolitica* exhibited similar inhibition zones (8.33–9.67 mm) despite differences in MIC_50_ values (13.6–15.9 mg/mL). Likewise, *Candida* species displayed nearly identical, small inhibition zones (5.33–6.33 mm), while MIC_50_ values varied considerably (25.5–41.4 mg/mL). Similar discrepancies between disc diffusion and dilution methods have been reported for essential oils and plant extracts, particularly when testing complex natural mixtures [52, 53].

The limited correspondence between disc diffusion and MIC results for Gram‐negative bacteria and fungi is likely related to differences in the diffusion behavior of hydrolate constituents in agar media. Hydrolates comprise complex mixtures of water‐soluble and partially water‐soluble compounds with diverse molecular sizes and polarities, which may diffuse at different rates through agar [54]. Furthermore, interactions between hydrolate components and agar polysaccharides may reduce the availability of active compounds at the agar surface, thereby affecting inhibition zone formation [55]. The hydrophobic nature of monoterpenes like thymol further complicates diffusion through aqueous agar matrices, resulting in concentration gradients that do not accurately reflect true antimicrobial potency [56].

These findings highlight an important methodological consideration: although the disc diffusion assay is useful for rapid, preliminary screening of antimicrobial activity, MIC determination by broth microdilution provides a more accurate and quantitative assessment of antimicrobial potency, particularly for complex natural mixtures such as plant hydrolates [57, 58]. Accordingly, future studies aimed at standardization and quality control of hydrolates should prioritize MIC‐based methods over disc diffusion assays. Statistical analysis revealed significant differences in antimicrobial response among the tested microorganisms (Tukey's HSD, *p* ≤ 0.05), suggesting differences in susceptibility as reflected in inhibition zone diameters and MIC values.

### 4.7 Comparison With Published Hydrolate Studies

Despite increasing interest in plant hydrolates, studies specifically addressing thymol‐rich thyme hydrolates remain limited, particularly in terms of comprehensive antimicrobial evaluation across different microbial groups. Direct comparison of the present results with previously published studies on plant hydrolates is limited by the relatively small number of comprehensive antimicrobial investigations in this area, particularly those focusing on thymol‐containing hydrolates. The majority of available studies primarily address essential oils rather than hydrolates, and when hydrolates are evaluated, they are frequently concentrated using solid‐phase extraction (SPE) or similar enrichment techniques prior to antimicrobial testing [16].

Available literature indicates that concentrated clove hydrolate (50‐fold SPE) exhibits MIC values of 0.4%–0.8% (equivalent to 4–8 mg/mL) against *S. aureus* and *E. faecalis*, with corresponding inhibition zones ranging from 15.5 to 18.8 mm [16]. In comparison, the non‐concentrated thymol hydrolate evaluated in the present study demonstrated MIC_50_ values of 5.765 mg/mL against *S. aureus* and 7.638 mg/mL against *E. faecalis*, with inhibition zones of 16.33 mm and 15.33 mm, respectively. These findings indicate that thymol hydrolate exhibits antimicrobial activity comparable to that reported for concentrated plant hydrolates, despite the absence of any concentration step.

The relatively high antimicrobial efficacy observed in this study can be attributed, at least in part, to the high thymol content (79.9%) of the hydrolate, which exceeds the thymol levels commonly reported for thyme essential oils (typically 30%–70%). The hydrodistillation process used for hydrolate production appears to have facilitated efficient transfer of thymol into the aqueous phase, resulting in a hydrolate with enhanced antimicrobial properties.

Only a limited number of studies have systematically evaluated the antimicrobial activity of hydrolates against both bacterial and fungal microorganisms [46]. In this context, the present work provides a broad assessment of antimicrobial efficacy by examining 14 microorganisms, including Gram‐positive bacteria, Gram‐negative bacteria, yeasts, and filamentous fungi. This comprehensive screening offers valuable comparative insight into the antimicrobial spectrum of thymol hydrolate and reveals a consistent susceptibility pattern (Gram‐positive bacteria > Gram‐negative bacteria > yeasts > filamentous fungi).

### 4.8 Hydrolates Versus Essential Oils: Practical Considerations

Although essential oils generally exhibit stronger antimicrobial activity than hydrolates, hydrolates offer several practical advantages that make them attractive for specific applications where moderate antimicrobial efficacy is sufficient. First, hydrolates contain substantially lower concentrations of volatile bioactive compounds than essential oils, resulting in a more favorable safety and tolerability profile. While essential oils may cause skin irritation, sensitization, or phototoxic reactions at higher concentrations, hydrolates are typically well tolerated and can often be applied directly to the skin without prior dilution [59]. This characteristic is particularly advantageous for cosmetic and personal care applications.

Second, hydrolates are inherently water‐based, which facilitates their incorporation into aqueous formulations such as toners, sprays, mists, and oral rinses. In contrast, essential oils require the use of emulsifiers or solubilizing agents for incorporation into water‐based systems, increasing formulation complexity and production costs [60]. Recent advances in nanoencapsulation technologies, including Pickering emulsions and nanoemulsions, have improved the stability and efficacy of essential oil‐based formulations [61, 62].

Third, hydrolates generally exhibit milder and more subtle aromatic profiles than essential oils. Their less intense fragrance makes them suitable for products where strong odors are undesirable, such as facial cosmetics, dermatological formulations, and products intended for sensitive users [46].

From an economic perspective, hydrolates are by‐products of essential oil distillation and are therefore typically more cost‐effective than essential oils [63]. This advantage is particularly relevant for applications requiring large volumes of antimicrobial agents, including agricultural treatments, industrial sanitation, or surface disinfection. The valorization of hydrolates contributes to the sustainability of essential oil production by reducing waste and enhancing the economic viability of aromatic plant cultivation.

Finally, hydrolates are commonly regulated as cosmetic or food‐grade ingredients rather than as pharmaceutical agents in many jurisdictions, which may simplify regulatory approval processes depending on the intended use. However, regulatory classification may vary across regions and applications, and proper quality control measures, including microbiological testing and preservation strategies, remain essential for ensuring product safety [59].

Taken together, these considerations suggest that thymol hydrolate may be suitable for applications in which moderate antimicrobial activity is adequate and where safety, ease of formulation, sensory properties, and cost‐effectiveness are prioritized over maximal antimicrobial potency.

### 4.9 Potential Applications

Based on the antimicrobial activity demonstrated in this study, thymol hydrolate may have potential applications in several fields where moderate antimicrobial efficacy, combined with favorable safety and formulation properties, could be advantageous.

In the context of food preservation, the pronounced activity against Gram‐positive bacteria, particularly *S. aureus* and *L. monocytogenes*, suggests that thymol hydrolate may be considered as a natural preservative or surface treatment for ready‐to‐eat foods. Recent studies have demonstrated the efficacy of thyme essential oil in reducing foodborne pathogens in meat products, with applications including edible coatings, nanoemulsions, and active packaging systems [8, 64, 65]. The use of bacterial cellulose/thyme essential oil emulsion coatings has been shown to effectively inhibit spoilage of chilled chicken during refrigerated storage [60], while pickering emulsion formulations have improved the stability and antimicrobial efficacy of thyme essential oil against foodborne pathogens [61]. However, efficacy in complex food matrices would require validation under practical conditions.

In cosmetics and personal care products, the observed antifungal activity against *Candida* species suggests the potential use of thymol hydrolate as a functional ingredient contributing to microbial stability and product safety. Applications may include water‐based formulations such as toners, facial mists, deodorants, and oral care products. The safety profile of hydrolates for long‐term skin use has been documented for several Lamiaceae species at appropriate concentrations [59]. In addition to antimicrobial properties, the presence of linalool may enhance consumer acceptability and provide additional sensory benefits [46].

With respect to medical and pharmaceutical applications, the activity observed against clinically relevant microorganisms, including *S. aureus*, *L. monocytogenes*, and *Candida* species, suggests potential utility in topical formulations, including wound care products or oral rinses. The synergistic antibacterial effects of thyme and clove essential oils have been demonstrated against food isolates of *E. coli* and *S. aureus* [8], supporting potential applications in infection control. Nevertheless, translation of these findings into medical use would require comprehensive in vivo efficacy studies, toxicological evaluation, and clinical validation before any therapeutic application could be considered.

In agriculture and horticulture, although antifungal activity against filamentous fungi was moderate, thymol hydrolate may be further investigated as a component of integrated pest management strategies, particularly in organic farming systems where the use of synthetic fungicides is restricted. Plant essential oils, including those from thyme, have gained increasing attention as biopesticides due to their target specificity, biodegradability, and safety for nontarget organisms [66, 67]. Recent advances in essential oil formulations, including nanoencapsulation and natural deep eutectic solvents (NaDES), have improved their stability and efficacy as biopesticides [66]. However, potential effects on beneficial microorganisms, such as *Trichoderma* species used in biological control, should be carefully evaluated.

Finally, recent studies reporting antifungal effects of thyme essential oils on paper substrates [4] suggest possible applications in the preservation of cellulose‐based materials. While the present study focused on food‐related and clinically relevant microorganisms, these findings point to broader potential uses of thymol hydrolate in the conservation of books, documents, and archival materials. However, further validation under real‐world conditions is required.

### 4.10 Limitations and Future Research Directions

Several limitations of the present study should be acknowledged. First, only a single reference strain of each bacterial and fungal species was evaluated. Future studies should include a broader range of strains, including clinical isolates and drug‐resistant variants, to better characterize the antimicrobial spectrum of thymol hydrolate and assess the potential for resistance development.

Second, the mechanisms underlying the antimicrobial activity of thymol hydrolate and possible interactions between thymol and other hydrolate constituents were not investigated. Further studies employing techniques such as transmission electron microscopy, membrane permeability assays, and transcriptomic or proteomic analyses could provide deeper insight into the modes of action involved.

Third, although MIC_50_ and MIC_90_ values were determined, minimum bactericidal and fungicidal concentrations (MBC/MFC) were not assessed, nor was activity against biofilm‐forming microorganisms evaluated. Since biofilms represent a major challenge in clinical, food‐processing, and industrial environments [68, 69], future research should focus on the ability of thymol hydrolate to inhibit biofilm formation or disrupt established biofilms. Recent studies have demonstrated that thyme essential oil and its major component, thymol exhibit significant antibiofilm activity against foodborne pathogens, including *Salmonella*, *E. coli*, *L. monocytogenes*, and *S. aureus* [70], suggesting that thymol hydrolate may possess similar properties that warrant investigation.

Fourth, the antimicrobial activity was evaluated exclusively under in vitro conditions. The efficacy of thymol hydrolate in real‐world applications may be influenced by environmental factors such as pH, temperature, organic matter, and interactions with complex matrices (e.g., food, skin, or packaging materials). Therefore, in situ and application‐specific studies are required to validate its practical effectiveness. Finally, the stability of thymol hydrolate under different storage conditions and over extended periods was not examined. Long‐term stability studies are essential to ensure consistent antimicrobial performance and shelf‐life suitability for commercial applications.

Future research should therefore focus on (i) evaluating synergistic effects between thymol hydrolate and other natural antimicrobials or reduced doses of conventional antimicrobial agents [71]; (ii) assessing the effects of sub‐inhibitory concentrations on microbial virulence factors and biofilm development; (iii) determining cytotoxicity and safety profiles for topical and oral applications; (iv) developing and optimizing delivery systems such as nanoemulsions, liposomes, or hydrogels to enhance antimicrobial efficacy; (v) conducting scale‐up and standardization studies for industrial production; and (vi) performing targeted in vivo and clinical studies for selected food, cosmetic, or medical applications.

### 4.11 Significance and Innovation

This study provides a comprehensive evaluation of a thymol chemotype *Thymus vulgaris* hydrolate against a diverse panel of bacteria, yeasts, and filamentous fungi using standardized quantitative antimicrobial assays. The high thymol content (79.9%) identified in the hydrolate represents a distinguishing compositional feature and contributes to the pronounced antimicrobial activity observed, particularly against Gram‐positive bacteria.

The results demonstrate that even a non‐concentrated thymol hydrolate exhibits antimicrobial activity comparable to that reported for a 50‐fold SPE‐concentrated clove hydrolate [16]. This observation underscores its potential practical relevance. Overall, thymol chemotype thyme hydrolate appears to be an effective plant‐derived antimicrobial option, particularly when factors such as safety, formulation simplicity, and cost‐effectiveness are considered.

More broadly, this work contributes to the limited but growing body of research on plant hydrolates and supports their potential as sustainable and economically viable alternatives to synthetic antimicrobial agents. In the context of increasing antimicrobial resistance [72, 73] and rising consumer demand for natural products [74, 75]. Atanasov et al. [76] and Guedes et al. [75], the present findings provide a valuable foundation for further research and development of hydrolate‐based antimicrobial solutions for food, cosmetic, and related applications.

## 5 Conclusion

This study provides a comprehensive evaluation of the antimicrobial activity of a thymol chemotype *Thymus vulgaris* L. hydrolate against a diverse panel of clinically and economically relevant microorganisms. HS‐GC–MS analysis revealed a high thymol content (79.9%), with linalool (17.4%) as the second major constituent, confirming the thymol chemotype and providing a chemical basis for the observed antimicrobial effects. The antimicrobial activity of thymol hydrolate followed a consistent susceptibility pattern: Gram‐positive bacteria > Gram‐negative bacteria > yeasts > filamentous fungi. Pronounced activity was observed against Gram‐positive pathogens, particularly *S. aureus*, *L. monocytogenes*, and *E. faecalis*, while Gram‐negative bacteria exhibited moderate susceptibility. Yeasts and filamentous fungi showed lower sensitivity, although measurable antifungal activity was still detected across all tested fungal species. Comparison of disc diffusion and broth microdilution methods demonstrated good agreement for Gram‐positive bacteria but limited correspondence for Gram‐negative bacteria and fungi, highlighting the importance of MIC‐based approaches for quantitative assessment of hydrolate antimicrobial activity. This methodological finding is relevant for future standardization and quality control of plant hydrolates. From a practical perspective, thymol hydrolate offers several advantages over essential oils, including improved safety and tolerability, water solubility, milder sensory properties, and potential cost‐effectiveness. These characteristics, combined with its demonstrated antimicrobial activity, suggest further investigation of thymol hydrolate as a potential natural antimicrobial agent for applications in food preservation, cosmetics, and related fields. Overall, this work contributes to the limited scientific knowledge on plant hydrolates and provides a basis for future studies focusing on the mechanism of action, in vivo efficacy, stability, and formulation development. Further research into synergistic combinations and activity against resistant microorganisms may enhance the applicability of thymol hydrolate in addressing current challenges related to antimicrobial resistance. However, further studies are required to confirm its effectiveness under practical conditions.

## Author Contributions

**Miroslava Kačániová**: conceptualization, data curation, writing – original draft, methodology, investigation, formal analysis, supervision, project administration, and funding acquisition. **Joel Horacio Elizondo‐Luévano**: review and editing, data curation, and formal analysis. **Anis Ben Hsouna**: data curation, writing – original draft, and formal analysis. **Rania Ben Saad**: data curation, writing – original draft, and formal analysis. **Stefania Garzoli**: conceptualization, formal analysis, methodology, data curation, review and editing, and supervision.

## Conflicts of Interest

The authors declare no conflicts of interest.

## Data Availability

The data that support the findings of this study are available from the corresponding author upon reasonable request.

## References

[1] H. M. Fathy , M. N. Ahmed , H. A. Goda , and M. A. Moselhy , “Thyme Essential Oil Potentials as a Bactericidal and Biofilm‐Preventive Agent Against Prevalent Bacterial Pathogens,” Scientific Reports 15 (2025): 31644. 40866486 10.1038/s41598-025-16485-5 PMC12391397

[2] C. Gan , E. Langa , A. Valenzuela , D. Ballestero , and M. R. Pino‐Otín , “Synergistic Activity of Thymol With Commercial Antibiotics Against Critical and High WHO Priority Pathogenic Bacteria,” Plants 12 (2023): 1868. 37176927 10.3390/plants12091868 PMC10180827

[3] S. D'Amato , A. Serio , C. C. López , and A. Paparella , “Hydrosols: Biological Activity and Potential as Antimicrobials for Food Applications,” Food Control 86 (2018): 126–137.

[4] F. Menicucci , A. Crisci , W. Tarraf , et al., “Exploring Wild *Thymus* sp. (L.) Chemotypes Across Pistoia Mountains Provides Thyme Essential Oil and Hydrolate Inhibiting Fungal Growth on Paper,” Fitoterapia 182 (2025): 106418. 39929393 10.1016/j.fitote.2025.106418

[5] A. Hajibonabi , M. Yekani , S. Sharifi , J. S. Nahad , S. M. Dizaj , and M. Y. Memar , “Antimicrobial Activity of Nanoformulations of Carvacrol and Thymol: New Trend and Applications,” OpenNano 13 (2023): 100170.

[6] S. F. Wirtu , K. Ramaswamy , R. Maitra , S. Chopra , A. K. Mishra , and L. T. Jule , “Isolation, Characterization and Antimicrobial Activity Study of *Thymus vulgaris* ,” Scientific Reports 14 (2024): 21573. 39284874 10.1038/s41598-024-71012-2 PMC11405399

[7] Z. Li , Y. Li , and W. Cheng , “Determination of Cinnamaldehyde, Thymol and Eugenol in Essential Oils by LC–MS/MS and Antibacterial Activity of Them Against Bacteria,” Scientific Reports 14 (2024): 12424. 38816435 10.1038/s41598-024-63114-8 PMC11139912

[8] D. Sateriale , G. Forgione , G. A. De Cristofaro , et al., “Antibacterial and Antibiofilm Efficacy of Thyme (*Thymus vulgaris* L.) Essential Oil against Foodborne Illness Pathogens, *Salmonella Enterica* subsp. Enterica Serovar Typhimurium and Bacillus Cereus,” Antibiotics 12 (2023): 485. 36978352 10.3390/antibiotics12030485 PMC10044538

[9] L. Hofmeisterová , T. Bajer , M. Walczak , and D. Šilha , “Chemical Composition and Antibacterial Effect of Clove and Thyme Essential Oils on Growth Inhibition and Biofilm Formation of Arcobacter spp. And Other Bacteria,” Antibiotics 13 (2024): 1232. 39766622 10.3390/antibiotics13121232 PMC11672449

[10] S. Garzoli , V. Laghezza Masci , S. Franceschi , A. Tiezzi , P. Giacomello , and E. Ovidi , “Headspace/GC–MS Analysis and Investigation of Antibacterial, Antioxidant and Cytotoxic Activity of Essential Oils and Hydrolates From *Rosmarinus officinalis* L. and Lavandula Angustifolia Miller,” Foods 10 (2021): 1768. 34441545 10.3390/foods10081768 PMC8392121

[11] J. D. Thompson , J.‐C. Chalchat , A. Michet , Y. B. Linhart , and B. Ehlers , “Qualitative and Quantitative Variation in Monoterpene Co‐Occurrence and Composition in the Essential Oil of *Thymus vulgaris* Chemotypes,” Journal of Chemical Ecology 29 (2003): 859–880. 12775148 10.1023/a:1022927615442

[12] E. Vassiliou , O. Awoleye , A. Davis , and S. Mishra , “Anti‐Inflammatory and Antimicrobial Properties of Thyme Oil and Its Main Constituents,” IJMS 24 (2023): 6936. 37108100 10.3390/ijms24086936 PMC10138399

[13] C. González‐Machado , C. Alonso‐Calleja , and R. Capita , “Methicillin‐Resistant *Staphylococcus aureus* (MRSA) in Different Food Groups and Drinking Water,” Foods 13 (2024): 2686. 39272452 10.3390/foods13172686 PMC11394615

[14] I. Habib , M.‐Y. I. Mohamed , G. B. Lakshmi , et al., “Prevalence, Antimicrobial Resistance, and Distribution of Toxin Genes in Methicillin‐Resistant *Staphylococcus aureus* From Retail Meat and Fruit and Vegetable Cuts in the United Arab Emirates,” Frontiers in Cellular and Infection Microbiology 15 (2025): 1628036. 41112579 10.3389/fcimb.2025.1628036 PMC12531260

[15] L. Xing , M. Cheng , S. Wang , et al., “Methicillin‐Resistant *Staphylococcus aureus* Contamination in Meat and Meat Products: A Systematic Review and Meta‐Analysis,” Frontiers in Microbiology 16 (2025): 1636622. 40735619 10.3389/fmicb.2025.1636622 PMC12303875

[16] D. Šilha , K. Švarcová , T. Bajer , et al., “Chemical Composition of Natural Hydrolates and Their Antimicrobial Activity on Arcobacter‐Like Cells in Comparison With Other Microorganisms,” Molecules 25 (2020): 5654. 33266263 10.3390/molecules25235654 PMC7730011

[17] A. Bolívar and F. Pérez‐Rodríguez , “Listeria in Food: Prevalence and Control,” Foods 12 (2023): 1378. 37048199 10.3390/foods12071378 PMC10093824

[18] J. C. C. P. Costa , A. Bolívar , T. M. Alberte , G. Zurera , and F. Pérez‐Rodríguez , “ *Listeria monocytogenes* in Aquatic Food Products: Spotlight on Epidemiological Information, Bio‐based Mitigation Strategies and Predictive Approaches,” Microbial Pathogenesis 197 (2024): 106981. 39349150 10.1016/j.micpath.2024.106981

[19] B. S. Das , A. Sarangi , I. Pahuja , et al., “Thymol as Biofilm and Efflux Pump Inhibitor: A Dual‐Action Approach to Combat *Mycobacterium tuberculosis* ,” Cell Biochemistry & Function 42 (2024): e70030. 39676255 10.1002/cbf.70030

[20] X. Wang , L. Tian , J. Fu , et al., “Evaluation of the Membrane Damage Mechanism of Thymol Against *Bacillus Cereus* and Its Application in the Preservation of Skim Milk,” Food Control 131 (2022): 108435.

[21] S. Shapiro and B. Guggenheim , “The Action of Thymol on Oral Bacteria,” Oral Microbiology and Immunology 10 (1995): 241–246. 8602337 10.1111/j.1399-302x.1995.tb00149.x

[22] F. Guo , Q. Chen , Q. Liang , et al., “Antimicrobial Activity and Proposed Action Mechanism of Linalool Against *Pseudomonas Fluorescens* ,” Frontiers in Microbiology 12 (2021): 562094. 33584604 10.3389/fmicb.2021.562094 PMC7875898

[23] R. He , W. Chen , H. Chen , et al., “Antibacterial Mechanism of Linalool Against *L. monocytogenes*, a Metabolomic Study,” Food Control 132 (2022): 108533.

[24] S.‐K. Yang , K. Yusoff , M. Ajat , et al., “Combinatorial Antimicrobial Efficacy and Mechanism of Linalool Against Clinically Relevant *Klebsiella pneumoniae* ,” Frontiers in Microbiology 12 (2021): 635016. 33815320 10.3389/fmicb.2021.635016 PMC8010000

[25] H. Nikaido , “Molecular Basis of Bacterial Outer Membrane Permeability Revisited,” Microbiology and Molecular Biology Reviews 67 (2003): 593–656. 14665678 10.1128/MMBR.67.4.593-656.2003 PMC309051

[26] P. Sperandeo , A. M. Martorana , and A. Polissi , Bacterial Cell Walls and Membranes, ed. A. Kuhn , (Springer International Publishing, 2019), 9–37.

[27] K. S. Pahil , M. S. A. Gilman , V. Baidin , et al., “A New Antibiotic Traps Lipopolysaccharide in Its Intermembrane Transporter,” Nature 625 (2024): 572–577. 38172635 10.1038/s41586-023-06799-7 PMC10794137

[28] S. Akermi , S. Smaoui , M. Fourati , et al., “In‐Depth Study of *Thymus vulgaris* Essential Oil: Towards Understanding the Antibacterial Target Mechanism and Toxicological and Pharmacological Aspects,” BioMed Research International 2022 (2022): 3368883. 35909468 10.1155/2022/3368883 PMC9334058

[29] A. S. Monsef , M. Nemattalab , S. Parvinroo , and Z. Hesari , “Antibacterial Effects of Thyme Oil Loaded Solid Lipid and Chitosan Nano‐Carriers Against *Salmonella* Typhimurium and *Escherichia coli* as Food Preservatives,” PLoS One 19 (2024): e0315543. 39739777 10.1371/journal.pone.0315543 PMC12140078

[30] S. E. Majowicz , J. Musto , E. Scallan , et al., “The Global Burden of Nontyphoidal *Salmonella* Gastroenteritis,” Clinical Infectious Diseases 50 (2010): 882–889. 20158401 10.1086/650733

[31] A. D. Teklemariam , R. R. Al‐Hindi , R. S. Albiheyri , et al., “Human Salmonellosis: A Continuous Global Threat in the Farm‐to‐Fork Food Safety Continuum,” Foods 12 (2023): 1756. 37174295 10.3390/foods12091756 PMC10178548

[32] European Food Safety Authority (EFSA) , European Centre for Disease Prevention and Control (ECDC) , ‘The European Union One Health 2023 Zoonoses Report’, EFS2 2024, 22, 10.2903/j.efsa.2024.9106. PMC11629028 39659847

[33] N. A. R. Gow and M. D. Lenardon , “Architecture of the Dynamic Fungal Cell Wall,” Nature Reviews Microbiology 21 (2023): 248–259. 36266346 10.1038/s41579-022-00796-9

[34] A. Ahmad , A. Khan , F. Akhtar , et al., “Fungicidal Activity of Thymol and Carvacrol by Disrupting Ergosterol Biosynthesis and Membrane Integrity Against *Candida* ,” European Journal of Clinical Microbiology & Infectious Diseases 30 (2011): 41–50. 20835742 10.1007/s10096-010-1050-8

[35] L. A. Walker , N. A. R. Gow , and C. A. Munro , “Elevated Chitin Content Reduces the Susceptibility of *Candida* Species to Caspofungin,” Antimicrobial Agents and Chemotherapy 57 (2013): 146–154. 23089748 10.1128/AAC.01486-12 PMC3535899

[36] A. Brand , “Hyphal Growth in Human Fungal Pathogens and Its Role in Virulence,” International Journal of Microbiology 2012 (2012): 1–11. 10.1155/2012/517529 PMC3216317 22121367

[37] G. K. K. Reddy , A. R. Padmavathi , and Y. V. Nancharaiah , “Fungal Infections: Pathogenesis, Antifungals and Alternate Treatment Approaches,” Current Research in Microbial Sciences 3 (2022): 100137. 35909631 10.1016/j.crmicr.2022.100137 PMC9325902

[38] Y. Lee , N. Robbins , and L. E. Cowen , “Molecular Mechanisms Governing Antifungal Drug Resistance,” npj Antimicrob Resist 1 (2023): 5. 38686214 10.1038/s44259-023-00007-2 PMC11057204

[39] S. Kauser , N. Raj , S. Ahmedi , and N. Manzoor , “Mechanistic Insight into the Membrane Disrupting Properties of Thymol in *Candida* Species,” The Microbe 2 (2024): 100045.

[40] P. C. Braga , M. Alfieri , M. Culici , and M. Dal Sasso , “Inhibitory Activity of Thymol Against the Formation and Viability of *Candida albicans* Hyphae,” Mycoses 50 (2007): 502–506. 17944714 10.1111/j.1439-0507.2007.01412.x

[41] N. A. R. Gow , J.‐P. Latge , and C. A. Munro , “The Fungal Cell Wall: Structure, Biosynthesis, and Function,” Microbiology Spectrum 5 (2017). 10.1128/microbiolspec.funk-0035-2016 PMC11687499 28513415

[42] N. P. Keller , “Fungal Secondary Metabolism: Regulation, Function and Drug Discovery,” Nature Reviews Microbiology 17 (2019): 167–180. 30531948 10.1038/s41579-018-0121-1 PMC6381595

[43] S. Fillinger and Y. Elad , eds., Botrytis – the Fungus, the Pathogen and Its Management in Agricultural Systems (Springer International Publishing, 2016).

[44] M. Hahn , “The Rising Threat of Fungicide Resistance in Plant Pathogenic Fungi: *Botrytis* as a Case Study,” J Chem Biol 7 (2014): 133–141. 25320647 10.1007/s12154-014-0113-1 PMC4182335

[45] D. J. Daferera , B. N. Ziogas , and M. G. Polissiou , “The Effectiveness of Plant Essential Oils on the Growth of *Botrytis cinerea, Fusarium* sp. and *Clavibacter Michiganensis* subsp. *Michiganensis* ,” Crop Protection 22 (2003): 39–44.

[46] H. H. S. Almeida , I. P. Fernandes , J. S. Amaral , A. E. Rodrigues , and M.‐F. Barreiro , “Unlocking the Potential of Hydrosols: Transforming Essential Oil Byproducts into Valuable Resources,” Molecules 29 (2024): 4660. 39407589 10.3390/molecules29194660 PMC11477756

[47] A. M. S. Al‐Hatmi , J. F. Meis , and G. S. De Hoog , “Fusarium: Molecular Diversity and Intrinsic Drug Resistance,” Plos Pathogens 12 (2016): e1005464. 27054821 10.1371/journal.ppat.1005464 PMC4824402

[48] S. L. Woo , M. Ruocco , F. Vinale , et al., “Trichoderma‐based Products and Their Widespread Use in Agriculture,” TOMYCJ 8 (2014): 71–126.

[49] J. Meletiadis , J. W. Mouton , J. F. G. M. Meis , and P. E. Verweij , “ *In Vitro* Drug Interaction Modeling of Combinations of Azoles With Terbinafine Against Clinical *Scedosporium prolificans* Isolates,” Antimicrobial Agents and Chemotherapy 47 (2003): 106–117. 12499177 10.1128/AAC.47.1.106-117.2003 PMC149034

[50] N. P. Wiederhold , Antifungal Drug Resistance, ed. D.J. Krysan and W.S. Moye‐Rowley (Springer, 2023), 3–16.

[51] E. C. on A. S. EUCAST, Breakpoint Tables for Interpretation of MICs and Zone Diameters, version, 2025.

[52] M. Balouiri , M. Sadiki , and S. K. Ibnsouda , “Methods for In Vitro Evaluating Antimicrobial Activity: A Review,” Journal of Pharmaceutical Analysis 6 (2016): 71–79. 29403965 10.1016/j.jpha.2015.11.005 PMC5762448

[53] C. Valgas , S. M. D. Souza , E. F. A. Smânia , and A. Smânia Jr. , “Screening Methods to Determine Antibacterial Activity of Natural Products,” Brazilian journal of microbiology 38 (2007): 369–380.

[54] I. Wiegand , K. Hilpert , and R. E. W. Hancock , “Agar and Broth Dilution Methods to Determine the Minimal Inhibitory Concentration (MIC) of Antimicrobial Substances,” Nature Protocols 3 (2008): 163–175. 18274517 10.1038/nprot.2007.521

[55] A. Klančnik , S. Piskernik , B. Jeršek , and S. S. Možina , “Evaluation of Diffusion and Dilution Methods to Determine the Antibacterial Activity of Plant Extracts,” Journal of Microbiological Methods 81 (2010): 121–126. 20171250 10.1016/j.mimet.2010.02.004

[56] K. A. Hammer , C. F. Carson , and T. V. Riley , “Effects of *Melaleuca alternifolia* (Tea Tree) Essential Oil and the Major Monoterpene Component Terpinen‐4‐ol on the Development of Single‐ and Multistep Antibiotic Resistance and Antimicrobial Susceptibility,” Antimicrobial Agents and Chemotherapy 56 (2012): 909–915. 22083482 10.1128/AAC.05741-11 PMC3264233

[57] CLSI, M07 | Methods for Dilution Antimicrobial Susceptibility Tests for Bacteria That Grow Aerobically, can be found under https://clsi.org/shop/standards/m07/, 2024.

[58] P. Cos , A. J. Vlietinck , D. V. Berghe , and L. Maes , “Anti‐infective Potential of Natural Products: How to Develop a Stronger In Vitro Proof‐of‐concept,” Journal of Ethnopharmacology 106 (2006): 290–302. 16698208 10.1016/j.jep.2006.04.003

[59] K. Smiljanić , I. Prodić , S. Trifunovic , et al., “Multistep Approach Points to Compounds Responsible for the Biological Activity and Safety of Hydrolates From Nine Lamiaceae Medicinal Plants on Human Skin Fibroblasts,” Antioxidants 12 (2023): 1988. 38001841 10.3390/antiox12111988 PMC10669667

[60] Y. Xu , J. Xin , Y. Lyu , and C. Zhang , “Effects of Bacterial Cellulose/Thyme Essential Oil Emulsion Coating on the Shelf Life of Chilled Chicken Meat,” Journal of the Science of Food and Agriculture 104 (2024): 5577–5587. 38372374 10.1002/jsfa.13392

[61] Y. Cahyana , Y. S. E. Putri , D. S. Solihah , F. S. Lutfi , R. M. Alqurashi , and H. Marta , “Pickering Emulsions as Vehicles for Bioactive Compounds From Essential Oils,” Molecules 27 (2022): 7872. 36431978 10.3390/molecules27227872 PMC9693335

[62] L. Liu , K. D. Fisher , and W. D. Bussey , “Comparison of Emulsion Stabilizers: Application for the Enhancement of the Bioactivity of Lemongrass Essential Oil,” Polymers 16 (2024): 415. 38337303 10.3390/polym16030415 PMC10857636

[63] L. Predná and M. Habánová , “Antioxidant Potential in Selected Species of Small Berry Fruits,” Acta fytotechn zootechn 18 (2015): 116–118.

[64] M. Posgay , B. Greff , V. Kapcsándi , and E. Lakatos , “Effect of *Thymus vulgaris* L. Essential Oil and Thymol on the Microbiological Properties of Meat and Meat Products: A Review,” Heliyon 8 (2022): e10812. 36247140 10.1016/j.heliyon.2022.e10812 PMC9562244

[65] S. Ricardo‐Rodrigues , M. I. Rouxinol , A. C. Agulheiro‐Santos , M. E. Potes , M. Laranjo , and M. Elias , “The Antioxidant and Antibacterial Potential of Thyme and Clove Essential Oils for Meat Preservation—An Overview,” Applied Biosciences 3 (2024): 87–101.

[66] I. Dridi , A. Landoulsi , N. Smirani , and Jonas , Biochemistry (IntechOpen, 2024).

[67] I. Gupta , R. Singh , S. Muthusamy , et al., “Plant Essential Oils as Biopesticides: Applications, Mechanisms, Innovations, and Constraints,” Plants 12 (2023): 2916. 37631128 10.3390/plants12162916 PMC10458566

[68] J.‐S. Kim , M. S. I. Khan , T.‐Y. Kim , and M.‐C. Lim , “Antibiofilm Properties of Essential Oils against Foodborne Bacteria: A Review of Mechanisms,” Food Science and Biotechnology (2025), 10.1007/s10068-025-01988-8. PMC13022165 41909871

[69] P. Yang , Y. Huo , Q. Yang , F. Zhao , C. Li , and J. Ju , “Synergistic Anti‐Biofilm Strategy Based on Essential Oils and Its Application in the Food Industry,” World Journal of Microbiology and Biotechnology 41 (2025): 81. 40011295 10.1007/s11274-025-04289-8

[70] S. Vidaković Knežević , S. Knežević , D. Milanov , et al., “Essential Oils as a Novel Anti‐Biofilm Strategy against *Salmonella* Enteritidis Isolated from Chicken Meat,” Microorganisms 13 (2025): 2412. 41156870 10.3390/microorganisms13102412 PMC12565988

[71] B. Saoudi , K. Bariz , S. Saci , et al., “Enhancing Antibiotic Efficacy and Combating Biofilm Formation: Evaluating the Synergistic Potential of *Origanum vulgare* Essential Oil Against Multidrug‐Resistant Gram‐Negative Bacteria,” Microorganisms 12 (2024): 1651. 39203493 10.3390/microorganisms12081651 PMC11356740

[72] M. Miethke , M. Pieroni , T. Weber , et al., “Towards the Sustainable Discovery and Development of New Antibiotics,” Nature Reviews Chemistry 5 (2021): 726–749. 10.1038/s41570-021-00313-1 PMC8374425 34426795

[73] T. M. Uddin , A. J. Chakraborty , A. Khusro , et al., “Antibiotic Resistance in Microbes: History, Mechanisms, Therapeutic Strategies and Future Prospects,” Journal of Infection and Public Health 14 (2021): 1750–1766. 34756812 10.1016/j.jiph.2021.10.020

[74] C. C. Danta , S. S. Gurav , and R. V. Chikhale , Antidiabetic Drug Discovery From Natural Products (Elsevier, 2025), 39–50.

[75] B. N. Guedes , K. Krambeck , A. Durazzo , et al., “Natural Antibiotics Against Antimicrobial Resistance: Sources and Bioinspired Delivery Systems,” Brazilian journal of microbiology 55 (2024): 2753–2766. 38888693 10.1007/s42770-024-01410-1 PMC11405619

[76] A. G. Atanasov , S. B. Zotchev , V. M. Dirsch , et al., “Natural Products in Drug Discovery: Advances and Opportunities,” Nature Reviews Drug Discovery 20 (2021): 200–216. 33510482 10.1038/s41573-020-00114-z PMC7841765

